# Burden of migraine related to menses: results from the AMPP study

**DOI:** 10.1186/s10194-015-0503-y

**Published:** 2015-03-18

**Authors:** Jelena M Pavlović, Walter F Stewart, Christa A Bruce, Jennifer A Gorman, Haiyan Sun, Dawn C Buse, Richard B Lipton

**Affiliations:** Department of Neurology, Albert Einstein College of Medicine, Bronx, NY USA; Montefiore Headache Center, Bronx, NY USA; Sutter Health, Concord, CA USA; Geisinger Clinic, Center for Health Research, Danville, PA USA

**Keywords:** Migraine, Disability, Burden, Prevalence, Menstrual cycle, Menstrual migraine

## Abstract

**Background:**

Studies of the difference between menstrually associated and non-menstrually associated migraine are somewhat controversial. The majority of studies have focused on comparing menstrual to non-menstrual attacks rather than comparing study groups with different migraine diagnoses with respect to menstruation. As there is limited knowledge available on the overall impact and burden of migraine among groups of women with and without menstrually associated migraine our goal was to examine differences between these groups. We hypothesized that there would be greater burden of migraine related to menstruation and headache frequency in a population study across groups of women.

**Methods:**

We analyzed data from the American Migraine Prevalence and Prevention (AMPP) Study, a longitudinal, US, population-based study. We included female respondents to the 2009 survey, aged 18 to 60, who met modified ICHD-2 criteria for migraine, were actively menstruating and fit one of three definitions based on the self-reported association of menses and migraine attacks: self-reported predominantly menstrual migraine (MM, attacks that only or predominantly occur at the time of menses), self-reported menstrually-associated migraine (MAM, attacks commonly associated with menses, but that also occur at other times of the month), and self-reported menstrually-unrelated migraine (MUM). These three groups were compared on characteristics and measures of headache impact and burden (Headache Impact Test– 6 item (HIT-6) and Migraine Disability Assessment Scale (MIDAS).

**Results:**

There were 1,697 eligible subjects for this study in the following categories: MM (5.5%), MAM (53.8%), or MUM (40.7%). Women with MM had an older age of migraine onset. Those with predominantly menstrually-related attacks (MM) had fewer headache-days but appeared to be more impaired by attacks. HIT-6 and MIDAS scores were significantly higher for both the MM and MAM groups compared with the MUM groups; however, effects were more robust for MM than MAM.

**Conclusions:**

Nearly 60% of women with migraine reported an association between migraine and menses. These women reported greater headache impact and migraine-related burden on functioning than those in whom migraines were not related to menstruation. Women with MM were more impaired by attacks while women with MAM had overall highest burden, likely due to experiencing migraines on additional days.

## Background

Many women with migraine report an association between migraine attacks and menses. These attacks have been referred to as “menstrual” migraine by both patients and clinicians and have been the focus of an evolving definition of Menstrual Migraine by the International Classification of Headache Disorders (ICHD-2) [1,2]. The proportion of women with menstrually-related migraine varies among studies from 3% [3] to 76% [4], depending upon study populations and diagnostic criteria used. The use of broad encompassing case criteria may mask a severity effect, especially if the effect is confined to a more narrowly defined menstrual migraine subgroup. Clinical experience and results from clinic based studies support that peri-menstrual migraine attacks are more severe [3,5-7], longer in duration [3,8-10], have greater associated impairment [9,10] and are more difficult to treat [3,8,10] than migraine attacks at other times of the month.

There has been a long-standing preconception that this severity of menstrually associated migraines shown in clinical studies has not been seen in population-based studies [11-13]. The common explanations for this have been the selection bias in specialty clinics and the outcomes that were measured to represent dimensions of severity (e.g., pain intensity, pain duration, symptoms, disability, etc). Lack of precise menstrual migraine diagnoses and frequent variability in interpretation of the diagnoses among researchers has added to the confusion. Furthermore, the majority of prior population and clinic-based studies comparing menstrual to non-menstrual migraine have been based on prospective diaries and have focused on comparing menstrual attacks to non-menstrual attacks [6,10,11,14,15], rather than comparing different groups of migraineurs [3]. Therefore, it was the goal of this study to examine the severity and associated burden/impact of migraine across groups that are variously affected by menstruation, rather than only across attacks. The diagnostic criteria for menstrual migraine are still evolving and remain in the appendix of the ICHD-3beta [2] with the requirement for prospective documentation of the association of migraine attack and menses with three months of diary data. Since no population study has obtained three month menstruation related diary data on more than a hundred women, there is value in examining subgroups of women based on their self-reported relationship of migraine to menstruation as commonly occurs in clinical settings.

In the present study, we used self-reported data from the 2009 American Migraine Prevalence and Prevention (AMPP) Study [16] to define three groups of women with migraine using criteria and definitions that are different, but in spirit consistent with the ICHD criteria [2] (Table 1): self-reported predominantly menstrual migraine (MM) for headaches that occur with menses (two days before to three days after the start of menstruation), self-reported menstrually-associated migraine (MAM) for headaches associated with menses, but that also occur at other times of the month, and self-reported menstrually-unrelated migraine (MUM). We then compared these three groups to determine if there was variation in the impact (Headache Impact Test– 6 item [HIT-6]) and migraine-related disability (Migraine Disability Assessment Scale [MIDAS]). We hypothesized that the association with menses will contribute to significant burden of migraine and that women with MAM will experience the most significant burden because they have menstrually-related attacks as well as attacks outside of the menstrual window.

**Table 1:** **Headache and menstrual cycle definitions**

**American Migraine Prevalence and Prevention Study, 2009**	
**Self-reported definitions used in this paper**	
Self-reported predominantly Menstrual migraine (MM)	Headaches in relation to your period: “a*ll of my headaches are related to my menstrual cycle*”(my headaches ONLY happen around the time of my period (from two days before to three days after the start of menstruation)
Self-reported Menstrually-associated migraine (MAM)	Headaches in relation to your period: “*I am more likely to get headaches with my period but they also occur at other times of the month*”
Self-reported Menstrually-unrelated migraine (MUM)	Headaches in relation to your period: “*my headaches are not related to my menstrual cycle*”
**International Headache Society (ICHD-3beta)** [2]	
**Diagnostic Criteria**	
Pure menstrual migraine	Documented and prospectively collected evidence that attacks occur exclusively on day 1 ± 2 (i.e., days -2 to +3)^1^ of menstruation in at least two out of three menstrual cycles and at no other times of the cycle
Menstrually-related migraine	Documented and prospectively collected evidence that attacks occur on day 1 ± 2 (i.e., days -2 to +3)^1^ of menstruation in at least two out of three menstrual cycles and additionally at other times of the cycle
Non-menstrual migraine	Attacks have no menstrual relationship

1- The first day of menstruation is day 1 and the preceding day is day -1; there is no day 0.

## Methods

The AMPP Study was approved by the Albert Einstein College of Medicine Institutional Review Board. The AMPP Study was modeled on the methods of the American Migraine Studies I [17] and II [18], described in detail elsewhere [16] and briefly summarized herein. In 2004, a screening survey was mailed to a stratified random sample of 120,000 U.S. households encompassing 257,339 household members [16]. The questionnaire was completed by the head of the household, who reported the total number of household members and the number of household members experiencing at least occasional self-defined “severe headache”. Each household member with severe headaches was asked to complete a questionnaire that included questions the AMS/AMPP diagnostic module which allows for assignment of migraine based upon on modified ICHD-2 criteria. This module has been demonstrated to have a sensitivity of 100% and specificity of 82% for the diagnosis of migraine [19]. No significant changes occurred between ICHD-2 and ICHD-3beta that are related to the criteria used in this study.

A random sample of 24,000 of the adult (i.e., ≥18 years of age) respondents to the 2004 survey who self-reported active (i.e., past 12 months) “severe headache” were asked to participate in an annual longitudinal follow up study. They completed questionnaires annually from 2005-2009. We used the 2009 AMPP Study survey because complete data were available to describe subtypes of menstrual migraine and it included the HIT-6 (with paid permission for use) as well as the MIDAS.

**Figure 1:**
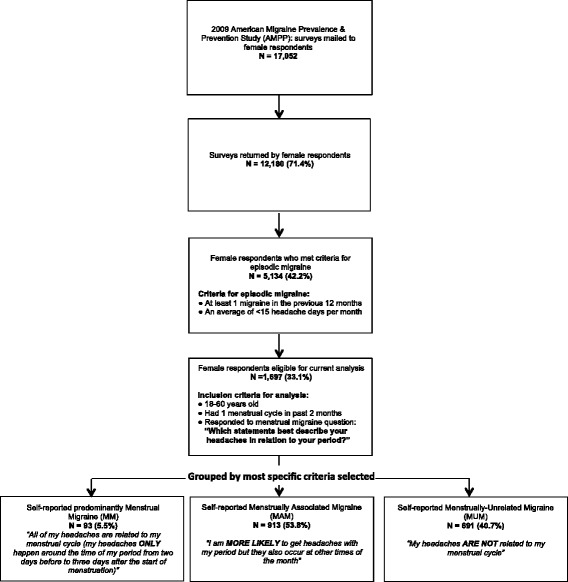
**2009 AMPP female respondents and group assignment.**

In the 2009 survey, respondents answered questions about headache symptoms, frequency, severity, menstrual status and whether they had headache/migraine associated with menstruation, employment status (i.e., working for pay full- or part-time, unemployed, retired, a student, a homemaker, disabled, a volunteer, on medical or maternity leave, or “other”) and completed the MIDAS [20] and HIT-6 questionnaire [21]. 17,052 females were mailed the 2009 AMPP Study questionnaire and 71.4% (n = 12,180) were completed and returned (Figure 1).

Respondents were asked to indicate the number of days they had headache in the preceding three months and the number of days with their “most severe type of headache” over the preceding year. A total of 5,134 female respondents of the 2009 AMPP Study survey met criteria for episodic migraine and were considered active cases (i.e., defined as having at least one migraine in the previous 12 months and an average of <15 headache days per month over the preceding 3 months). Of these, 1,697 were eligible for this analysis which included the following: aged 18 to 60 years, had at least one menstrual cycle in the preceding two months, and responded to the questions used to determine menstrual migraine status.

### Menstrual migraine subgroups

The diagnostic criteria for migraine in association with menses have not been firmly established; as a consequence in ICHD-3beta [2] (Table 1) the criteria remain in the Appendix, indicating that they are not yet fully accepted, but intended for further study. We could not apply ICHD-3beta criteria as written because we relied on self-reported recall of headaches associated with menses, a common approach both in large scale epidemiological studies and in clinical care. Our large scale study does not include daily diaries as recommended by ICHD-3beta criteria in order to establish a diagnosis.

The AMPP Study questionnaire asked, “*Which statements best describe your headaches in relation to your period?*” Respondents were classified into three mutually exclusive groups based on their responses. Women who selected “*all of my headaches are related to my menstrual cycle (my headaches ONLY happen around the time of my period from two days before to three days after the start of menstruation)*” were assigned to the self-reported predominantly menstrual migraine (MM) group. Women who selected “*I am MORE LIKELY to get headaches with my period but they also occur at other times of the month*” were assigned to the self-reported menstrually-associated migraine (MAM) group unless they also endorsed the MM statement. Finally, women who selected “*my headaches are NOT related to my menstrual cycle*” were assigned to the self-reported menstrually-unrelated migraine (MUM) group if they did not also endorse the MM or MAM statement. Another difference between how we defined our subgroups and ICHD-3beta criteria is that we did not require women to endorse “*I get headaches with at least two of every three periods*” as these data were not collected.

We did not exclude women who endorsed more than one response option. Of respondents who endorsed the response “*My headaches ONLY happen around the time of my period [from two days before to three days after the start of menstruation]*” about one third also endorsed “*I am MORE LIKELY to get headaches with my period but they also occur at other times of the month*”. Simultaneous endorsement of both statements most likely occurs in women who have predominantly MM with occasional migraine and/or headaches at other times. Accordingly, we assigned these women to the self-reported predominantly MM group (MM). This decision and its potential consequences are considered in the discussion. “*I am MORE LIKELY to get headaches with my period but they also occur at other times of the month*” and “*my headaches are NOT related to my menstrual cycle*” were both endorsed by 4.1% of the cases and these women were assigned into the MAM group. This is likely conservative since these cases likely decreased the strength of observed associations.

### Number of migraine attacks and days with migraine/headache

Headache attack frequency was based on a response to a question about the number of days with headache (for most severe type of headache) in the past month, three months, and 12 months and converted to a measure of headaches per month. Headache-days in the past three months was taken from the response to a MIDAS question on the number of days with headache in the past three months (one migraine attack could last for multiple days).

### Headache impact on functioning

We used the following migraine/headache impact measures: MIDAS (scored by grade), a MIDAS measure of lost productive time (LPT) [22,23] and the HIT-6 [21].

### Migraine-related disability

The MIDAS is comprised of five questions related to impact of migraine/headache on functioning in defined roles (i.e., housework, work for pay, leisure time) [20] that are used to categorize severity into four grades based on total days of impaired functioning (i.e., Grade I or little or no disability, 0 to 5; Grade II or mild disability, 6 to 10; Grade III or moderate disability, 11 to 20; Grade IV or severe disability, ≥21, with an option of dividing Grade IV into IVA and IVB). The recall period for the MIDAS is three months, effectively covering three menstrual cycles.

### Lost productive time

We used the two MIDAS work-for-pay questions to estimate lost productive time (LPT). These two questions ask about number of days of missed work in the past three months due to headache (absenteeism) and number of days while at work where productivity was reduced by half (presentism denoted as Half Days) or more because of headache.

### Employment

Employment status was documented [22] for each respondent and defined as actively employed for pay (i.e., excludes those on short- or long-term medical leave) if they reported actively working enough hours to qualify for full- (≥35 hours) or part-time (20 to 34 hours) status in the previous two weeks. Individuals were defined as eligible for employment but “unemployed” if they reported they were either unemployed or on medical disability. For analysis, employment was defined as a binary variable (i.e., employed versus unemployed).

We also used the HIT-6, (with a licensing fee) which is comprised of six questions, to assess the impact of the most severe headache type on ability to function. Three questions are independent of headache frequency and three questions are related to frequency of headache attacks. It has a recall period of four weeks [21]. It should be noted that both MIDAS and HIT-6 impact measures do not specifically assess headache or migraine days related to menstruation, but all headache-days over an interval of 3 months and 1 month, respectively.

### Analyses

The analysis was confined to women who both met criteria for migraine and had menstrual cycles in the two-month period before completing the 2009 AMPP Study survey. We compared the three subgroups defined by self-reported menstrual headache status (i.e., MM, MAM, MUM) on characteristics and measures of headache impact.

LPT was defined as: Missed Work Days + 0.5*(Workdays Where Productivity was Reduced by Half)

The LPT formula is for the number of missed workday equivalents in the three months before the survey. We assumed that a workday reduced by half or more is equivalent to working for half of a usual workday, an estimate that was validated in a previous diary study [20]. Based on previous experience [22,23] and given the non-normal distribution properties of work loss (i.e., a substantial proportion with no work loss), LPT was defined as two binary variables (i.e., < 0.5 LPT Days versus 0.5+ LPT days and < 2 LPT Days versus 2+ LPT days). For analysis, employment was defined as a binary variable (i.e., employed versus unemployed). We applied the standard scoring algorithm for HIT-6 items where responses are assigned values (i.e., Never = 6, Rarely = 8, Sometimes = 10, Very Often = 11, Always = 13) and summed for a total score. We stratified respondents using the standard cut-points. (i.e., <50 = little or no impact, and 50 to 55 for some impact, 56 to 59 for substantial impact, ≥60 for very severe impact). We completed regression analysis using cut-points above and below 56 (i.e., <56, ≥56) and 60 (i.e., <60, ≥60).

We used chi-square to compare the sociodemographic and headache characteristics of the three subgroups, and we used logistic regression (i.e., LPT, HIT-6 item variables) to analyze the relationship between menstrual migraine status and measures of headache impact, as previously defined. We completed separate logistic regression models for women who reported six or fewer headache-days in the past three months (i.e., an average of two headache-days per month) and for all women with EM (i.e., ≤45 headache-days in three months). Covariates were added in two sequential models that included: 1) age (18 to 29, 30 to 39, 40 to 49, 50 to 60); race (White, Black, Other); education (no high school diploma, high school diploma, some college or associates degree, bachelors degree, graduate degree); annual household income (< $22,500, $22,500to $39,999, $40,000 to $59,999, $60,000 to $89,999, ≥ $90,000), and age at migraine onset; and 2) number of attacks of the most severe type of headache in the past month. SAS version 9.2 was used for all analyses (SAS Institute Inc., Cary, NC).

## Results

Among the 1,697 respondents who met inclusion criteria (Table 2), 5.5% (n = 93) were categorized as MM, 53.8% (n = 913) as MAM, and 40.7% (n = 691) as MUM using the criteria presented in Table 1. Overall, the three groups significantly differed by education, annual household income, number of headache types, and age of onset of migraine. Those in the MAM group tended to have a higher education level, whereas those in the MM group tended to have a higher income. Women with MM had fewer headache types and a later age of migraine onset (i.e., 34.4% on or after age 30 versus <24.0% for the other two groups). Menstrual migraine association status was not associated with other covariates. The number of headache-days in the past three months differed significantly among the three groups (Table 3). In the MM group, 68.8% of cases had six or fewer headache-days in the past three months compared to 53.3% for MAM cases and 61.7% for MUM cases (p < 0.001). In the MAM group, persons with ≥15 headache-days in the past three months were more common (p < 0.001). The distribution by number of headache attacks is difficult to compare by group because of the variation in percent with unknown values. In particular, 17.2% of MM cases had an unknown value. Furthermore, the difference in distribution by number of headache-days is an artifact, in part, of the case definition for MM (i.e., “*my headaches ONLY happen around the time of my period [from two days before to three days after the start of menstruation]*”).

**Table 2:** **Sociodemographic and headache characteristics by menstrual group in women with episodic migraine who are actively menstruating: results from the AMPP 2009 Survey**

	**All (N = 1,697)**		**Stratified by Menstrual Group**					
			**Self-reported predominantly Menstrual Migraine (MM) (N = 93)**		**Self-reported Menstrually associated migraine (MAM) (N = 913)**		**Self-reported Menstrually unrelated migraine (MUM) (N = 691)**	
**Age, years**								
<30	196	11.6%	10	10.7%	103	11.3%	83	12.0%
30-39	554	32.6%	22	23.7%	289	31.6%	243	35.2%
40-49	748	44.1%	44	47.3%	410	44.9%	294	42.6%
50-60	199	11.7%	17	18.3%	111	12.2%	71	10.3%
**Race**								
White	1495	88.1%	77	82.8%	811	88.8%	607	87.8%
Black	118	7.0%	11	11.8%	59	6.5%	48	6.9%
Asian, Pacific Islander	28	1.6%	2	2.2%	13	1.4%	13	1.9%
Other	27	1.6%	1	1.1%	17	1.9%	9	1.3%
Unknown	29	1.7%	2	2.2%	13	1.4%	14	2.0%
**Education** ^**1**^								
High school grad or less	375	22.1%	25	26.9%	176	19.3%	174	25.2%
Some college	412	24.3%	22	23.7%	216	23.7%	174	25.2%
Bachelor degree	639	37.6%	29	31.2%	373	40.8%	237	34.3%
Graduate degree	240	14.1%	14	15.1%	133	14.6%	93	13.5%
Unknown	31	1.8%	3	3.2%	15	1.6%	13	1.9%
**Current Employment Status** ^**2**^								
Full-time	958	56.2%	47	50.5%	520	57.2%	391	56.6%
Part-time	269	16.1%	22	23.7%	154	16.9%	93	13.5%
Unemployed	248	14.3%	13	14.0%	113	12.4%	122	17.7%
Other	218	13.2%	11	11.8%	122	13.4%	85	12.3%
**Annual Household Income** ^**3**^								
<$22,500	248	14.6%	16	17.2%	108	11.8%	124	17.9%
$22,500-$39,999	321	18.9%	16	17.2%	176	19.3%	129	18.7%
$40,000-$59,999	403	23.7%	18	19.4%	225	24.6%	160	23.2%
$60,000-$89,999	331	19.5%	16	17.2%	196	21.5%	119	17.2%
≥$90,000	394	23.2%	27	29.0%	208	22.8%	159	23.0%
**Body Mass Index**								
Underweight	81	4.8%	1	1.1%	45	4.9%	35	5.1%
Normal	571	33.6%	27	29.0%	328	35.9%	216	31.3%
Overweight	431	25.4%	28	30.1%	233	25.5%	170	24.6%
Obese	614	36.2%	37	39.8%	307	33.6%	270	39.1%
**Headache Types** ^**4**^								
1	455	26.8%	42	45.2%	225	24.6%	188	27.2%
2	772	45.5%	32	34.4%	432	47.3%	308	44.6%
≥3	413	24.3%	12	12.9%	232	25.4%	169	24.5%
Unknown	57	3.4%	7	7.5%	24	2.6%	26	3.8%
**Age of Onset – most severe headache** ^**5**^								
<15	351	20.7%	13	14.0%	218	23.9%	120	17.4%
15-19	316	18.6%	15	16.1%	182	19.9%	119	17.2%
20-24	272	16.0%	5	5.4%	143	15.7%	124	17.9%
25-29	190	11.2%	11	11.8%	97	10.6%	82	11.9%
≥30	384	22.6%	32	34.4%	192	21.0%	160	23.2%
Unknown	184	10.8%	17	18.3%	81	8.9%	86	12.5%

1 – Chi -square p = 0.035 for education level, when include unknowns p = 0.064.

2 – Chi-square p = 0.019 for employment status; “Unemployed” encompasses reported unemployed and those on medical leave. “Other” includes homemaker, retired, student, volunteer and other. Self-employed was included in the full time employed group.

3 – Chi-square p = 0.028 for household income.

4 – Chi-square p < 0.001 for headache type (including/excluding unknowns).

5 – Chi-square p = 0.001 for age of onset of most severe headache type (including/excluding unknowns).

**Table 3:** **Headache experience and impact by menstrual group: results from the AMPP 2009 survey**

**Measure**	**All (N = 1,697)**		**Stratified by menstrual group**					
			**Self-reported predominantly Menstrual Migraine (MM) (N = 93)**		**Self-reported Menstrually associated migraine (MAM) (N = 913)**		**Self-reported Menstrually unrelated migraine (MUM) (N = 691)**	
**Headache-Days in the Past 3 months (MIDAS item)** ^**1**^								
0-3	605	35.6%	37	39.8%	271	29.7%	297	43.0%
4-6	371	21.9%	27	29.0%	215	23.6%	129	18.7%
7-10	299	17.6%	12	12.9%	180	19.7%	107	15.5%
11-14	88	5.2%	4	4.3%	57	6.2%	27	3.9%
≥15	306	18.0%	10	10.7%	179	19.6%	117	16.9%
Unknown	28	1.6%	3	3.2%	11	1.2%	14	2.0%
**Headache Attack Frequency in the Past Month** ^**2**^								
0 – <1	568	33.5%	13	14.0%	279	30.6%	276	39.9%
1 – <2	563	33.2%	44	47.3%	324	35.5%	195	28.2%
2 – <3	214	12.6%	10	10.7%	130	14.2%	74	10.7%
≥3	260	15.3%	10	10.7%	149	16.3%	101	14.6%
Unknown	92	5.4%	16	17.2%	31	3.4%	45	6.5%
**MIDAS Grade** ^**3**^								
**I.** Little or no disability	1057	62.3%	54	58.1%	533	58.4%	470	68.0%
**II.** Mild disability	247	14.6%	16	17.2%	145	15.9%	86	12.5%
**III.** Moderate disability	184	10.8%	9	9.7%	122	13.4%	53	7.7%
**IV.** Severe disability	154	9.1%	6	6.4%	95	10.4%	53	7.7%
Unknown	55	3.2%	8	8.6%	18	2.0%	29	4.2%
**Lost Productive Time (LPT) (days in past 3 months) (MIDAS item)** ^**4**^								
0	760	61.9%	34	49.3%	404	59.9%	322	66.5%
≤1.00	182	14.8%	10	14.5%	99	14.7%	73	15.1%
1.01 – 4.99	167	13.6%	12	17.4%	111	16.5%	44	9.1%
≥5	59	4.8%	5	7.2%	37	5.5%	17	3.5%
Unknown	59	4.8%	8	11.6%	23	3.4%	28	5.8%
**Sum Score for 3 HIT-6 Questions** ^**5**^								
≤49: little to no impact	263	15.5%	15	16.1%	123	13.5%	125	18.2%
50-55: some impact	378	22.4%	17	18.3%	181	19.9%	180	26.2%
56-69: substantial impact	295	17.5%	9	9.7%	165	18.1%	121	17.6%
≥64: very severe impact	754	44.6%	52	55.9%	441	48.5%	261	38.0%

1- Chi-square tests p < 0.001 for MIDAS headache-days in past 3 months with or without unknown values.

2- Chi-square tests p < 0.001 for HA frequency in past month with or without unknown values.

3- Chi-square p < 0.001 for MIDAS grade with or without unknown values.

4- Chi-square p < 0.001 for MIDAS LPT with or without unknown values; restricted to full time or part time employed group (n = 1,227).

5- Chi-square tests p < 0.001for HIT-6 sum score.

### Headache impact

Impact measures, which were dependent on headache-days, also differed by menstrual migraine status. Specifically, the distribution by the four MIDAS Grades varied significantly by menstrual migraine status (chi-square p < 0.001). Proportionately more MAM (23.8%) and MM (16.1%) cases had MIDAS scores which classified them as Grade III or IV (moderate or severe) compared to MUM (15.4) cases. The unemployment rate (i.e., looking for work but not employed/[looking for work + employed]) differed (p = 0.019), with the highest rate occurring among MM cases (17.7%) compared to the other two groups (12.4% to 14.0%). In univariate analysis, only distribution by the MIDAS LPT measure differed among the three migraine groups (p < 0.001) (Table 3).

The three migraine subgroups differed by age, headache-days, and other factors that could confound the comparison on measure of functional impact. We completed logistic regression models (i.e., comparing MM and MAM cases to MUM cases) using measures of headache impact as the outcome for respondents with six or fewer headache-days in the past three months (58.5% of all cases and 68.8% of MM cases) and then for all EM cases (headache-days frequency per three months <45) (Table 4). The odds ratios did not differ for those with six or fewer headache-days/three months compared to all EM cases. We also adjusted for all covariates except for headache attack frequency (Model I) and then again by including headache attack frequency (Model II). The odds ratios were similar for models without (Model I) and with (Model II) adjustment for headache attacks in the previous month. Among those with six or fewer headache-days/three months (model 1), statistically significant elevated odds ratios were observed for MIDAS LPT (OR = 2.4, 95% CI: 1.1-5.1) and for a HIT-6 score of 56+ (OR = 2.5; 95% CI: 1.3-4.7) among MM cases (p < 0.01). Odds ratios for MAM cases were consistently lower than that of MM cases and were less often statistically significant.

**Table 4:** **Adjusted odds ratios (logistic regression) and associated confidence intervals for headache impact measures comparing the two menstrual migraine groups to the menstrually-unrelated migraine group, adjusting for potential confounders in two separate models** ^**1,2**^

**Outcome**	**Group** ^**3**^	**Headache-Days Frequency (per 3 months)**			
		**<=6**		**<=45**	
		**MODEL**		**MODEL**	
		**I** ^**1**^	**II** ^**2**^	**I** ^**1**^	**II** ^**2**^
Odds Ratio for MIDAS LPT^4^ (0.5+ Days vs <0.5 Days)^5^	MM	***2.4 (1.1-5.1)*** ^***7***^	2.0 (0.9-4.3)	***2.1 (1.1-3.8)*** ^***7***^	***1.9 (1.0-3.5)*** ^***7***^
	MAM	1.3 (0.9-1.9)	1.2 (0.8-1.9)	**1.5 (1.2-2.0)** ^**6**^	**1.5 (1.1-1.9)** ^**6**^
Odds Ratio for MIDAS LPT^4^ (2+ Days vs <2 Days)^5^	MM	**5.4 (1.8-15.7)** ^**6**^	**5.1 (1.7-15.3)** ^**6**^	***2.6 (1.2-5.5)*** ^***7***^	***2.7 (1.2-5.7)*** ^***7***^
	MAM	1.8 (0.9-3.7)	1.6 (0.8-3.4)	**1.9 (1.3-2.9)** ^**6**^	**1.8 (1.2-2.6)** ^**6**^
Odds ratio for HIT-6 sum score of 56+ vs <56^8^	MM	***2.5 (1.3-4.7)*** ^***7***^	1.7 (0.9-3.3)	***2.0 (1.1-3.3)*** ^***7***^	***1.9 (1.1-3.3)*** ^***7***^
	MAM	**1.4 (1.1-1.9)** ^**6**^	1.3 (0.9-1.8)	**1.5 (1.2-1.9)** ^**6**^	**1.4 (1.1-1.8)** ^**6**^
Odds ratio for HIT-6 sum score of 60+ vs <60^9^	MM	**4.0 (2.1-7.3)** ^**6**^	**2.9 (1.5-5.5)** ^**6**^	**3.0 (1.8-4.9)** ^**6**^	**2.9 (1.7-5.1)** ^**6**^
	MAM	***1.4 (1.0-1.9)*** ^***7***^	1.4 (0.9-1.9)	**1.6 (1.3-2.0)** ^**6**^	**1.5 (1.2-2.0)** ^**6**^

1-Includes age, race, education level, household income and age of migraine onset as covariates.

2- Includes the covariates from footnote “1” and headache attack frequency per month.

3- MM = self-reported predominanty menstrual migraine, MAM = self-reported menstrually-associated migraine, MUM = self-reported menstrually- unrelated migraine.

4-Lost Productive Time.

5-Restricted modeling: removed ‘other’ employed and MIDAS LPT models only includes those full time or part time employed.

6- Indicates migraine group significance using Type III test at P-value <0.01.

7-Indicates migraine group significance using Type III test at P-value <0.05.

8 – This cut-score divides individuals with some impact or less from those with substantial impact or more.

9 – This cut-score divides individuals with substantial impact or less from those with very severe impact or more.

In bold, values of statistical significance.

## Discussion

To assess burden of migraine in the population, we compared three groups of women with migraine based on the self-reported association of their migraine attacks with menses using data from the AMPP Study. The majority of women reported some association of migraine with menses, though in most, migraine also occurred at other times of the month. Those with predominantly menstrually-related attacks (MM) had fewer headache-days but appeared to be more impaired by attacks as measured by impairment in ability to work and elevated HIT-6 scores. Women with MAM had overall highest burden, due to experiencing not only peri-menstrual migraines but additional migraines outside of the peri-menstrual window.

Women with MM had fewer headache-days per three months, as was expected given the case definition for MM. In contrast, the MAM group had the highest number of headache-days per three months. This pattern may be important when interpreting findings for the MIDAS LPT headache-days adjusted measure and the HIT-6 measure (Table 4). These measures reflect, in part, the frequency of headaches, as well as, the impact that headaches have on functioning. The adjusted odds ratios for MIDAS LPT were statistically significant and indicate that women with MM experience headaches that have a substantial impact on the work role that is more disabling than that of the other two subgroups. Moreover, as the threshold for work impact was increased (i.e., missed days of work or absenteeism), the difference between MM cases and other cases increased, a finding that is consistent with specialty clinic [9,10] and population studies [3,24]. The HIT-6 measures the global impact of headaches on the social role and other domains of functioning and includes a measure of pain. The elevated odds ratios for a “severe impact” HIT-6 score (≥56) is consistent with the above findings for MIDAS and suggests a more general impact on functioning from MM than the work role.

Previous diary studies compared all peri-menstrual headaches to headaches at other times in the cycle. The prior study from our group based on prospectively filled diaries from the general population [11] and one clinic study suggested that attacks of menstrual and nonmenstrual migraine have similar disability [25]. However, other studies primarily from specialty clinics, suggest that attacks of peri-menstrual migraine are more disabling than attacks of nonmenstrual migraine [8-10]. All these studies focused on comparisons of specific migraine attacks rather than migraine groups.

In this study women with any of the menstrually-related migraine disorders reported on *all* of their headaches, rather than separately reporting on the impact of menstrually-related and menstrually-unrelated migraine. If menstrually-related migraine attacks are more disabling, then our data will underestimate impact of these headaches. The extent to which impact is underestimated will depend on the ratio of the number of menstrually-related migraine to the number of migraine occurring at other times in the cycle. This “dilution” effect is likely to be less significant for MM cases. The overall self-assessment by MM cases may largely represent experience with peri-menstrual headaches in contrast to the MAM and MUM groups who are largely reporting on non-peri-menstrual headaches. It is of interest that one third of our MM cases also endorsed that they have headaches outside of their menstruation (-2 to +3 days from start on menstruation window), further diluting the MM subgroup and the likely burden of migraine in those who have only menstrual migraines. As clinical experience suggests these are likely women who do not meet ICHD diagnostic criteria for pure MM, but likely experience the majority of their attacks during the menstrual window and only rare attacks at other times. As both clinic and population diary studies have been limited to three months, it is uncertain whether women who fulfill diagnostic criteria for pure MM in these studies actually occasionally experience attacks outside of their menstrual window. Clinical experience suggests so, but further longitudinal studies are needed to clarify this. Furthermore, if peri-menstrual attacks are truly more severe and burdensome as evidence suggests, there would be a bias in reporting of these events compared to non-menstruation related events. As such, the findings from this study cannot be used to determine if peri-menstrual headaches among women with MM, MAM or MUM are more disabling than headaches that occur at other times in the cycle. However, there is a clear trend of overall greater burden in the MM group than in MAM and both have greater burden than the MUM.

This study has several strengths. First, the study population was population-based and was not subject to the potential selection bias that is a concern with clinic-based samples. That is, our study population was less likely to be prone to selection bias that can occur if women are more likely to seek care because they have migraine headaches associated with menses that are not responsive to treatment. Second, the study population (N = 1,697) was large and representative of the demographics of the U.S. population. Finally, we used well validated instruments to assign migraine diagnosis and asses our primary variables of headache-impact and migraine-related disability.

The primary limitation in this study is that our operational criteria for assigning women to different subgroups differed from the ICHD recommended definitions. Diary data over several months would have been the ideal means of classifying women. Since these data were not available, we relied on self-reported, retrospective data. Our data likely mirror the information that patients will provide to healthcare professionals in clinical practice unless the patient or healthcare professional gather three months of prospective daily diary data. Nonetheless, we assume that self-identification of menstrual migraine status resulted in some misclassification and we acknowledge that we are not approximating the ICHD criteria for menstrual migraine. Rather, this is a descriptive study of women in the US population describing their perception of the relationship between their migraine attacks and their menstrual cycle. Notably, almost a third of MM cases reported seven or more headache-days in the past three months. It is likely that a number of these women did not have pure MM according to ICHD, although it is possible if their headaches last three days or if they also have non-migraine headaches. Furthermore, misclassification of MAM to MM cases is likely to have occurred as outlined above and this would diminish the strength of observed associations (i.e. biased the odds ratio toward the null) because data from MAM would have been falsely mixed in with the MM data. In contrast, misclassification of MM cases as MAM or MUM is likely to have had a negligible effect on assessing differences in disability due to the small size of the group in comparison to other groups.

Furthermore, our definition for MAM (“*I am more likely to get headaches with my period but they also occur at other times of the month*”) was broad and did not require that migraine occur only two days prior or three days after the start of menstruation as is required by ICHD criteria. Individuals could have responded yes to having a “menstrually-associated migraine” even if their migraine occurred on the final three days of their period, which does not match menstrual migraine criteria. It will be useful in the future to determine if peri-menstrual headaches in the MAM group are more disabling than headaches that occur at other times in the cycle. We also did not limit our EM group to those without aura, as is listed in ICHD criteria. Finally, menstrually related migraine has the benefit of being relatively predictable which allows for the preemptive use of therapy or short-term prophylaxis for migraine prevention. While short-term prophylaxis or preemptive use of triptans is not FDA approved, it is a strategy that is used in clinical practice for patients with menstrual related migraine [8,26,27]. In the current study we did not report or control for short-term prophylaxis; therefore it is possible that some percentage of the MM group may be well controlled on this therapeutic strategy. Therefore, the disability seen among that group may be reduced due, in some part, to short-term prophylaxis.

## Conclusion

In this large population study representative of the US population, we have shown that most women self-report migraine attacks associated with menstruation and those attacks are associated with impairment including impact on occupational, academic and household responsibilities. When these women are divided into subgroups, those with predominantly peri-menstrual migraines experience the greatest burden. While suggestions for treatment strategies may help, more research will be required to better understand these subgroups given that they account for a significant majority of women with migraine and to determine the best course of treatment for MM and MAM cases and as a result, reduce the burden of migraine associated with menstruation.

## Abbreviations

AMPP: American migraine prevalence and prevention study
MM: Self-reported predominantly menstrual migraine
MAM: Self-reported menstrually associated migraine
MUM: Self-reported menstrually unrelated migraine
ICHD: International classification of headache disorders
MIDAS: Migraine disability assessment scale
HIT: Headache impact test
PMM: Pure menstrual migraine
MRM: Menstrually related migraine
EM: Episodic migraine
LPT: Lost productive time
